# Dietary ergot alkaloids as a possible cause of tail necrosis in rabbits

**DOI:** 10.1007/s12550-014-0208-0

**Published:** 2014-09-19

**Authors:** A. K. Korn, M. Gross, E. Usleber, N. Thom, K. Köhler, G. Erhardt

**Affiliations:** 1 Department of Animal Breeding and Genetics, Justus-Liebig-University, Ludwigstrasse 21b, 35390 Giessen, Germany; 2 Institute of Veterinary Food Science, Dairy Sciences, Justus-Liebig-University, Ludwigstrasse 21, 35390 Giessen, Germany; 3 Clinic for Small Animals, Internal Medicine, Justus-Liebig-University, Frankfurter Strasse 126, 35392 Giessen, Germany; 4 Institute of Veterinary Pathology, Justus-Liebig-University, Frankfurter Strasse 96, 35392 Giessen, Germany

**Keywords:** Rabbits, Tail necrosis, Feed, Mycotoxins, Ergot alkaloids, *Fusarium* toxins

## Abstract

This study describes the association between tail necrosis in rabbits and mycotoxins in rabbit feed. Clinical cases of tail necrosis were observed in 14 out of 103 rabbits kept in an outdoor group housing, fed with hay and a commercial pelleted feed. The observed clinical symptoms, alopecia, erosions, crusts and necrosis were restricted to the tail area and exclusively occurred in young rabbits aged 113 ± 20 days. Dermatological examination suggested that ischemia had caused necrosis. Analysis of blood samples showed an elevated level of creatine kinase. No weight loss occurred in affected rabbits. Trauma caused by injuries or technopathic lesions was also excluded. Histopathologically, the lesions were characterized by acute muscle fibre degeneration and chronic active dermatitis with granulation tissue formation. Necropsy of one rabbit revealed hepatocellular degeneration and necrosis as remarkable findings. Feed analysis for ergot alkaloids by enzyme immunoassays yielded a mean and maximum ergot alkaloid content of 410 ± 250 μg/kg and 1,700 μg/kg, respectively. Faeces of affected rabbits contained ergot alkaloids at levels up to 200 μg/kg. The mean and maximum dietary intake of total ergot alkaloids were 17 and 71 μg/kg bodyweight, respectively. *Fusarium* toxins (trichothecenes, zearalenone, fumonisins) were also found in the feed, but at levels which did not explain the observed effects. The results indicate that ergot alkaloids may have been the cause of tail necrosis, which is supported by literature data showing that rabbits are especially sensitive towards these toxins.

**Electronic supplementary material:**

The online version of this article (doi:10.1007/s12550-014-0208-0) contains supplementary material, which is available to authorized users.

## Introduction

Mycotoxins are a challenge in everyday agricultural practice as there is fungal growth that subsequently causes mycotoxicosis in about 25 % of the world’s crop (CAST Task Force Report 2003), depending on environmental factors like the climate as well as genetic contributions of the plants themselves (Fink-Gremmels 1999). Mycotoxicosis due to incorporation of various mycotoxins in the feed is known in all farm animals, especially horses (Caloni and Cortinovis 2011), pigs (Colvin et al. 1993; Harvey et al. 1990; Kanora and Maes 2009; Pang et al. 1986; Weissenbacher-Lang et al. 2012) and poultry (Shareef 2010) but also has been described in pet animals such as in guinea pigs (Carlton and Tuite 1970). Due to the modern agricultural methods, acute intoxications in livestock animals are rare, while subacute or chronic progression is more common (Charmley et al. 1995). Adverse effects depend on the type of toxic agent, the concentration and the time of exposure (Fink-Gremmels 1999). Hepatic necrosis, haemorrhage, icterus (Caloni and Cortinovis 2011), severe skin lesions like ear necrosis (Weissenbacher-Lang et al. 2012), myocardial and pancreatic lesions (Pang et al. 1986) are found. Especially swine are considered as very sensitive to these effects (Kanora and Maes 2009). In Europe, *Fusarium* toxins (trichothecenes, zearalenone, fumonisins) are regarded as the most important mycotoxins with regard to animal health. For example, fumonisins lead to pulmonary edema, thoracic effusions and hepatocellular necrosis (Colvin et al. 1993). In cattle, experimental dosing of T-2 toxin caused necrosis of the tail tips, respectively red and encrusted lesions, when administered orally or intramuscularly. Furthermore, a severe intestinal haemorrhage could be found in necropsy (Grove et al. 1970).

Ergotism caused by *Claviceps purpurea* is the oldest known mycotoxicosis and is characterized by peripheral vasoconstriction followed by ischemic acral necrosis in cattle, goat, sheep and a number of other livestock species. After being almost neglected over the past decades, ergot and ergot alkaloids have regained scientific interest in recent years (Bennett and Klich 2003; Ginn et al. 2007; EFSA 2005; EFSA 2012). Very few information is available concerning adverse effects of ergot alkaloids in rabbits. To the best of our knowledge, only one reference gives some specific information on toxicity data for ergotamine and ergometrine in rabbits (Griffith et al. 1978). These authors report that rabbits are very sensitive towards ergot alkaloids, although no detailed experimental data are given in this publication (Table 1).

**Table 1:** LD_50_ values for different ergot alkaloids in rabbits, rats and mice after intravenous (i.v.) or oral application (data from Griffith et al. 1978)

Alkaloid	Rabbit		Rat		Mouse	
	LD_50_ (mg/kg body weight)					
	i.v.	oral	i.v.	oral	i.v.	oral
Ergonovine (−metrine)	3.2	27.8	120	671	160	460
Ergotamine	3.0	550.0	38	1300	265	3200
Ergocornine	0.9	n.s.	95	>500	275	2000
α-Ergocryptine	1.0–0.8	n.s.	140	n.s.	275	n.s.
Ergocristine	1.9	n.s.	64–150	n.s.	110	n.s.

*n.s.* not specified

Several authors (for example, Filipov et al. 1998; Panaccione et al. 2006) reported adverse nutritional effects of ergot alkaloids in rabbits, such as feed avoidance or reduced weight gain. These studies referred to ergovaline, which is an ergot alkaloid produced by endophytic fungi, but not by *Claviceps* spp., in grasses such as *Lolium perenne* and *Festuca arundinaceae*. However, ergovaline seems to have distinctively different toxicological properties compared with ergot alkaloids produced by *Claviceps* spp. Furthermore, the extracts used in these studies contained mixtures of ergovaline and other clavine alkaloids or even other classes of toxins; therefore, an unambiguous assignment of the effects is difficult.

With the exception of fumonisins, no specific regulations naming maximum levels of mycotoxins in rabbit feed exist. The European Commission recommended that the level of fumonisins in feed should not exceed 5 mg/kg. Other feed recommendations include deoxynivalenol (DON), zearalenone, T-2 toxin/HT-2 toxin and ochratoxin A (European Commission 2006; European Commission 2013), but these values are not based on specific toxicity data from studies with rabbits. A maximum level of 1,000 mg/kg of rye ergot sclerotia has been established for feedingstuffs containing unground cereals by European Union directive 2002/32/EC (European Union 2002). However, this regulation has limitations with regard to toxicity of feed because (1) the alkaloid content of ergot is variable, (2) sclerotia with different alkaloid pattern may occur not only in rye but also in other cereals and (3) sclerotia cannot reliably be determined in processed feed.

For example, ergot of rye sclerotia normally has an alkaloid content of 0.03–0.1 % (Appelt and Ellner 2009; Franzmann et al. 2011), but this value may go up to 1.5 % under favourable conditions (Hulvova et al. 2013). Consequently, the European Commission recently recommended that more data on rye ergot and ergot alkaloids in food and feed should be collected, with the intention to obtain reliable data on the ergot alkaloid pattern in feed and food and to relate the presence of ergot alkaloids to the amount of sclerotia present (European Commission 2012). It is however foreseeable that commercial rabbit feed will not play an important role in such a monitoring programme.

In this paper, we report a case study of spontaneous tail necrosis in a rabbit colony. Griffith et al. (1978) have described tail gangrene in rats after intraperitoneal injection of ergotoxin (ergocristine + ergocryptine + ergocornine). Peripheral ischemia after intravenous dosing of ergot alkaloids in several species, due to vasoconstriction, has also been reported by these authors. Although the clinical picture of ergot alkaloid intoxication in rabbits has not been described before, we therefore followed the hypothesis that ergot alkaloids could have been the causative agents, or at least a contributing factor, for the clinical observations.

## Materials and methods

### Animals and husbandry

A total of 103 rabbits were kept in an outdoor facility at the teaching and research unit (Oberer Hardthof) of Justus Liebig University Giessen, in groups of seven to nine animals per cage on a perforated plastic floor. Male and female animals were housed in separate compartments. They also had the possibility to withdraw in a wooden box, spread with shavings and straw. The stocking rate and the availability of space for each animal were in accordance with European Union directive 63/2010 (European Union 2010).

Ethical statement: The animals were originally kept for a breeding experiment approved by the local animal welfare authorities (Regierungspräsidium Giessen; Gi 17/11 No. 65/2011). The study was carried out according to the German regulations with regard to animal welfare.

Animals were fed with hay and a commercial pelleted feed preparation ad libitum and had unrestricted access to water from a nipple drinker. The composition and the analytical constituents of the pelleted feed are given in Table S1 (Supplementary material). After first tail lesions became visible and as soon as the feed was suspected to be a possible cause of the disease, the feed from batch 1 was withdrawn and submitted to mycotoxin analyses. For follow-up analyses, pelleted feed (batches 2 and 3) of the same provider was then fed to the animals and consecutively tested for mycotoxins as well.

All rabbits in the colony were regularly vaccinated against myxomatosis (Cunivak Myxo, IDT Biologika GmbH, Dessau-Rosslau, Germany) and rabbit haemorrhagic disease (Cunivak RHD, IDT Biologika GmbH, Dessau-Rosslau, Germany) according to manufacturers’ specifications.

### Clinical observations

The first rabbits with clinical symptoms of tail lesions were observed on 12 November 2012. The last observed case was on 07 January 2013. The mean age of the rabbits when showing first clinical signs was 113 ± 20 days. Until then, no pathological findings were observed. Feed and management factors were consistent since the start of the original breeding experiment. The daily feed consumption was assessed by weighing rabbits of two different groups and feed daily over a 1-week period as basis to calculate the amount of mycotoxin which was incorporated per kilogram bodyweight. Also, four healthy adult rabbits of corresponding breeds were included in this procedure to compare their dietary intake per kilogram bodyweight with that of the affected growing animals. All affected rabbits were weighted (Platform scales DE 60K20N, Kern & Sohn GmbH, Balingen-Frommern) up to four times at a 1-week interval, starting when the first lesions became apparent.

In total, 14 rabbits, which were offspring of four different breeding pairs, were clinically affected, and all of these affected rabbits were included in this study. Two unaffected animals served as controls for the blood parameters.

To rule out behavioural abnormalities and injuries caused by group mates, the rabbits were observed in several intervals during the day (for 10 min every 2 h) between 0700 hours (morning) and 1700 hours (afternoon). Cages and housing equipment were checked for possible sources of technopathic lesions.

Every affected animal underwent a complete general clinical examination. The physiological range was determined according to Schall (2008).

Blood samples were collected from three affected (82050, 82061, 82063) and two unaffected (82053, 82076) animals and analysed for clinical chemistry (Pentra 400, Horiba, Germany and Nova CRT 8, Nova Biomedical GmbH, Roedermark, Germany) and haematology (ADVIA 120, Siemens Healthcare, Eschborn, Germany).

Three rabbits (82050, 82059, 82063) underwent a detailed dermatological examination 9 days after clinical lesions were visible.

To avoid secondary infections, animals with ulcerative lesions received antibiotic treatment with enrofloxacin (Baytril 2.5 % solution, 0.4 ml per kg body weight, subcutaneous injection; Bayer AG, Leverkusen, Germany) for 5 days; additionally, chlortetracycline was applied topically (CTC-Blauspray, Novartis, Munich, Germany).

For two severely affected animals, tail amputation became necessary because of cranial progression of the lesions. Surgery was performed under ketamine hydrochloride/medetomidine anaesthesia (Haberstroh and Enke 2004). The amputated tails were then examined histopathologically. Tissue was fixed in 10 % neutral buffered formalin and embedded in paraffin using routine methods. Sections of about 4-μm thickness were stained with haematoxylin and eosin. One animal, which died 3 weeks after surgical intervention, was subjected to gross pathology and histopathology.

### Mycotoxin analyses

Feed samples taken from 44 bags, corresponding to three different batches of the same brand of commercial feed, were collected and analysed with competitive direct enzyme immunoassays (EIA, in-house methods) for total (generic) ergot alkaloids (GEA-EIA) and ergotamine (ergotamine-EIA). Additionally, four randomly selected samples of the second batch were analysed by an EIA for fumonisins. More *Fusarium* toxins, namely DON, T-2/HT-2 toxin and zearalenone (one sample from each batch) were also tested by EIAs. All tests were performed similarly as already described by Liesener et al. (2010). Furthermore, three feed samples from other producers, obtained from rabbitries without clinical signs of tail lesions, were analysed for total ergot alkaloids and for ergotamine.

Pooled faecal samples of four groups of animals (F1–F4; Table 2) were collected on five different days. Three of these series of samples (F1, F2, F4) were from affected groups of rabbits; the fourth series of samples (F3) was from a group of unaffected animals of a similar age from the research station (Table 2). Furthermore, samples of clinically symptom-free rabbits, obtained from two other husbandries (F5–F7; Table 2), were collected and also analysed for total ergot alkaloids and for ergotamine.

**Table 2:** Rabbit groups for faecal sampling

Group numbers	ID, animals in group	affected (×)/unaffected (−)
Groups from research station		
F1	82049 m/ 82060 f	×
F2	82059 f/ 82065 f	×
F3	82073 f/ 82074 f/ 82075 f/ 82076 f	−
F4	82052 f/ 82054 f/ 82063 f/ 82064 f	×
Groups from other husbandries		
F5	husbandry 1; 2 rabbits (1 m/1 f)	−
F6	husbandry 1; 2 rabbits (1 m/1 f)	−
F7	husbandry 2; 3 rabbits (3 f)	−

*m* male, *f* female

Additionally, samples of hay (three small bales, 7 kg each) which were fed to the rabbits and samples of straw (one 200-kg round bale) which were used as bedding were also analysed for ergot alkaloids. Macroscopically, no signs of mould were visible in these materials. From each dry material, a 300-g composite sample was collected, cut into pieces of 2- to 4-cm length and further ground to a mean particle size of <2 mm in a laboratory mill before analysis.

All EIAs were performed as standard microtiter plate tests. Sample preparation was performed as already described earlier (Liesener et al. 2010; Riemel 2012). At least three serial dilutions of all extracts were analysed. All extract dilutions resulting in absorbance values (B / B_0_ × 100) between 25 and 75 % were used to calculate the toxin content. Highly positive extracts, resulting in absorbance values outside the measuring range (<25 %), were further diluted and re-analysed by EIA.

## Results

### Clinical observations

The average daily feed intake of the affected (growing) rabbits was 42 g per kg body weight, the average feed intake of adult rabbits was 35 g per kg body weight.

The general clinical examination of all 14 affected rabbits yielded results within a normal range, body weight development was normal. Neither aberrant behavioural patterns within each group of rabbits nor indications for technopathic lesions were observed.

**Fig. 1:**
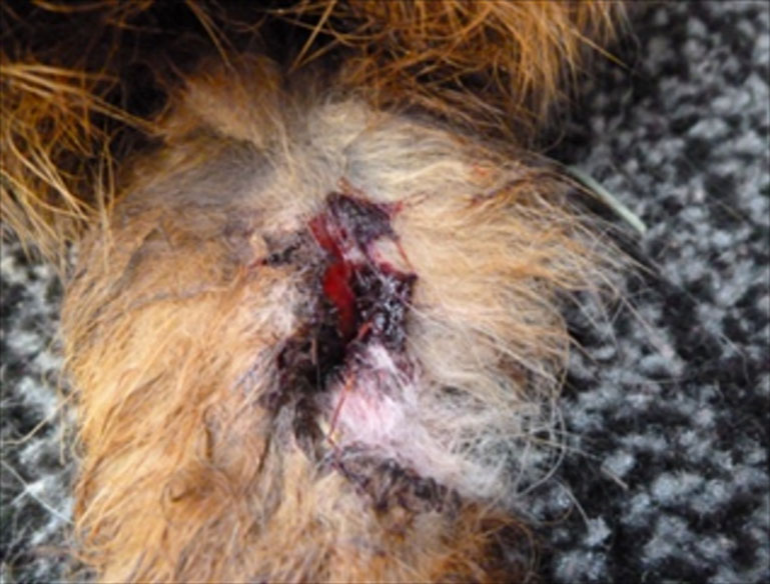
Tail region of one severely affected rabbit (82050) with sanguineous lesion. The lesion is located at the dorsal area and stretching over the last third of the tail

**Fig. 2:**
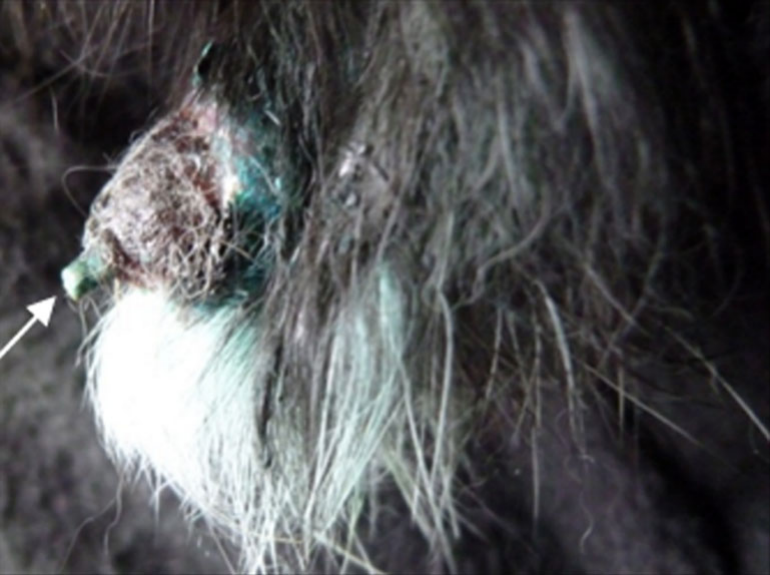
Tail region of one severely affected rabbit (82060) with necrotic lesion. The spine at the tail tip is visible (*arrow*), following necrosis of the skin tissue (*lateral view*)

Symptoms were first observed in November 2012 in six female rabbits (82050, 82052, 82059, 82060, 82061, 82063). All affected animals showed, to varying degrees, moist, sanguineous lesions at their tails, partly exposing the spine (Fig. 1) and affecting predominantly the vertebrae caudales at the distal part of the tail. About 2 days after the first symptoms were observed, necrotic alterations were visible, which progressed cranially and involved up to two thirds of the tail (Fig. 2).

**Fig. 3:**
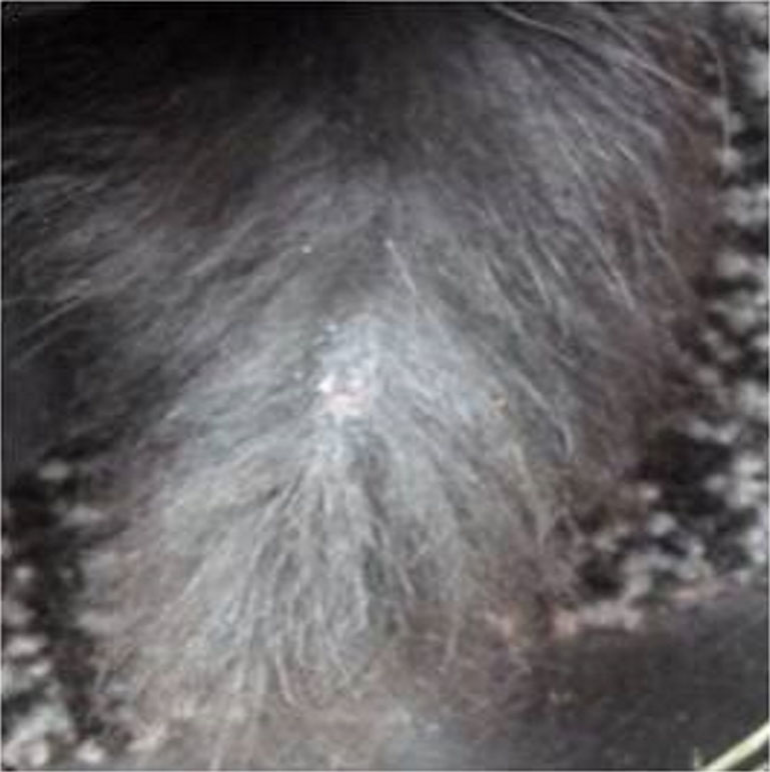
Tail region of one affected rabbit (82057) with fur loss. Mild skin alterations like alopecia and scaling are visible (*dorsal view*)

Eight more rabbits with symptoms of tail lesions were identified within the next 55 days, the last animal with symptoms was detected on 07 January 2013. Four of these rabbits (82054, 82057, 82064, 82066) had only mild skin alterations, such as alopecia and scaling (Fig. 3), the other four animals (82049, 82051, 82062, 82065) were more severely affected and showed crusted erosions and ulcerations of the tails.

The blood biochemistry profile (Table S2, Supplementary material) revealed through *t* test (95 % confidence interval) significantly higher creatine kinase values in the three affected rabbits ($\overline{x}$ = 1846.7 U/l) compared to the unaffected rabbits ($\overline{x}$ = 680.5 U/l). Levels of phosphorus and magnesium as well as alkaline phosphatase were slightly elevated both in affected and unaffected rabbits. Haematology results (Table S3, Supplementary material) were within physiological range.

Three rabbits (82050, 82059, 82063), which were presented for dermatological examination, all showed varying degrees of alopecia, scales, multifocal crusted erosions and ulcerations on the distal part of the tail. Other acra, namely the ears, were not affected. Slight scaling on the back, due to infestation with low numbers of fur mites (*Leporacarus gibbus*), was detected in one rabbit (82050). Differential diagnoses included ischemic dermatopathies (vasculopathy, vasculitis, toxic or cold-induced vasoconstriction), automutilation, barbering (trauma by cage mates), technopathic lesions and—less likely—dermatophytosis for cases with mild symptoms such as alopecia and scaling.

**Fig. 4:**
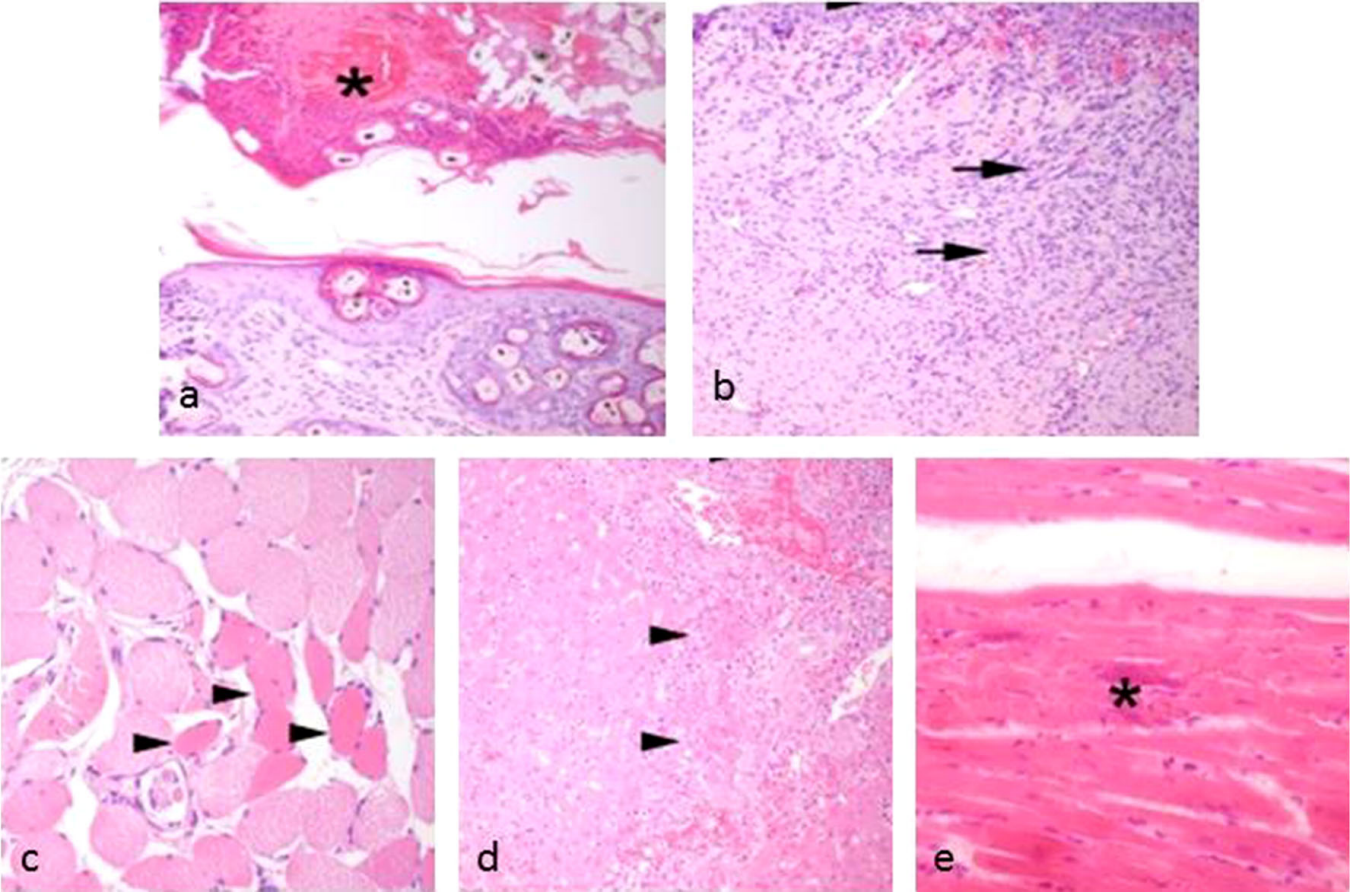
Histopathological findings in tail and organ tissue of affected rabbits. **a** Pyodermic tail. Please note the thick serocellular crust (*asterisk*) composed of degenerated leucocytes, keratin and blood cells (HE, ×20). **b** Skin beneath ulceration. The figure is showing epithelial hyperplasia (*arrowhead*) and chronic dermatitis with granulation tissue formation (*arrows*) (HE, ×20). **c** Degenerating muscle fibres. Hypereosinophilia and loss of myofibrils characterize degenerating muscle fibres of the tail in cross section (HE, ×20). **d** Histopathology of the liver of the necropsied animal (82050). Multiple foci of acute coagulative necrosis (*arrowhead*), bile duct hyperplasia, chronic cholangitis and pericholangitis (*arrow*) (HE, ×20) are shown. **e** Myocardial fibre necrosis. Acute myocardial fibre necrosis with dystrophic mineralization (*asterisk*) is visible in the heart of the necropsied animal (HE, ×40)

Histopathologically, the skin of the amputated tails was multifocally ulcerated with serocellular hemorrhagic crusts (Fig. 4a), hyperplasia of the surrounding epithelium (Fig. 4b) and melanin incontinence. Further, histopathological examination of tail tissue revealed an acute degeneration of single-striated muscle fibres (Zenkers degeneration) of the tail musculature (Fig. 4c), accompanied by regeneration. Other observations included distinct formation of granulation tissue in the subcutaneous tissue (Fig. 4b), single hair granulomas and prominent periosteal hyperplasia of the coccygeal vertebrae.

The deceased animal submitted for necropsy (82050) was in a good nutritional state. The liver was firm, irregularly mottled and showed multiple, sharply demarcated pale foci (diameter 0.5–1.0 cm), multifocally distributed within the parenchyma. The spleen was swollen due to acute congestion, and there was an acute diffuse alveolar edema in the lungs. Histopathologically, the liver showed multifocal areas of coagulative necrosis (Fig. 4d), randomly distributed with infiltration by neutrophils and macrophages. There was a mild anisocytosis and anisokaryosis of hepatocytes, and single binucleated cells were detected. Additionally, a marked chronic cholangitis and pericholangitis with fibrosis and bile duct hyperplasia (Fig. 4d), as well as an acute myofibril degeneration and necrosis with dystrophic mineralization in the myocardium, were observed (Fig. 4e). Other examined tissues and organs were without any remarkable lesions.

### Mycotoxin analyses

Mycotoxin analyses for *Fusarium* toxins by EIA yielded performance data similar as described previously (Liesener et al. 2010). For the total ergot alkaloid (TOT) EIA, the detection limit in rabbit feed was 50 ng/g, in rabbit faeces, it was 20 ng/g. Mean recoveries at spiking levels of 100–1,000 ng/g were 154–219 % in rabbit feed and 79–112 % in rabbit faeces. The detection limit of the ergotamine EIA in rabbit feed was 80 ng/g, in rabbit faeces, it was 30 ng/g. In rabbit feed and rabbit faeces spiked with ergotamine at levels of 100–1,000 ng/g, mean recoveries were 155–178 % and 92–336 %, respectively.

Total ergot alkaloids (TOT) could be detected in all 44 samples from three batches of the commercial rabbit feed, at a mean concentration of 410 ng/g (range 140–1,700 ng/g). Ergotamine (TAM) could also be detected in all samples, in a mean concentration range of 370 ng/g (range 140–910 ng/g). An overview of analytical results is given in Table 3. In feed samples of animal groups from husbandries without clinical symptoms, the concentration of total ergot alkaloids was 85–290 ng/g (mean 160 ng/g; *n* = 3), the concentration of ergotamine was 120–140 ng/g (mean 130 ng/g; *n* = 2).

**Table 3:** EIA results for total ergot alkaloids (TOT) and ergotamine (TAM) in rabbit feed

	Batch 1		Batch 2		Batch 3		Batches 1–3	
	TOT	TAM	TOT	TAM	TOT	TAM	TOT	TAM
n*	4	4	26	26	14	14	44	44
mean, μg/kg	570	650	400	370	370	290	410	370
SD, μg/kg	190	190	300	110	150	95	250	150
median, μg/kg	560	620	310	350	320	300	330	350
min, μg/kg	360	470	150	190	140	140	140	140
max, μg/kg	780	910	1700	760	680	430	1700	910

*Number of analysed samples from a total of 44 different 25-kg bags, three batches of feed from the same brand

Low levels of fumonisins could also be found in all four samples in a concentration range of 5.3–6.5 ng/g. All analysed samples, three from each feed batch, also contained DON (260–540 ng/g), T-2 toxin/HT-2 toxin (8.7-17 ng/g) and zearalenone (105–130 ng/g).

**Fig. 5:**
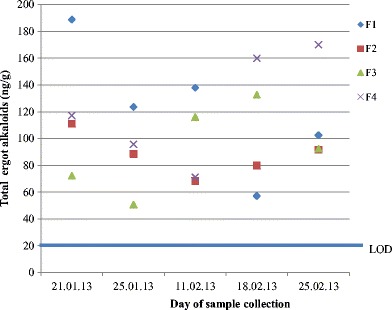
Time course of ergot alkaloids in rabbit faeces (total ergot alkaloid EIA results). Faecal samples were from four groups (F1, F2, F4 affected; F3 unaffected; see Table 2) from the research station were collected on five different days. The EIA limit of detection (LOD) was 20 ng/g. The average total ergot alkaloid concentration of all samples was 110 ng/g. Five faecal samples from three other groups (F5–F7) of rabbits from two other husbandries were all negative (<20 ng/g) for total ergot alkaloids

**Fig. 6:**
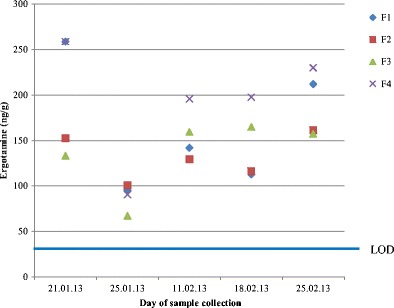
Time course of ergot alkaloids in rabbit faeces (ergotamine EIA results). Faecal samples from four groups (F1, F2, F4: affected; F3: unaffected; see Table 2) from research station were collected on five different days. The EIA limit of detection (LOD) was 30 ng/g. The average ergotamine concentration of all samples was 140 ng/g. When five faecal samples from three other groups (F5-F7) of rabbits from two other husbandries were analysed, two yielded weakly positive results close to the detection limit (30–40 ng/g)

Rabbit faeces samples both from the affected animal groups (F1, F2, F4) and from the unaffected group (F3) contained total ergot alkaloids in a concentration range of 51–180 ng/g (mean 110 ng/g, *n* = 20) (Fig. 5) and ergotamine in a concentration range of 44–260 ng/g (mean 140 ng/g, *n* = 20) (Fig. 6). The mean levels of groups F1, F2, F3 and F4 were 150, 93, 92 and 106 ng/g in the total ergot alkaloid EIA. The corresponding values in the ergotamine EIA were 194, 130, 136 and 146 ng/g. The five samples of faeces from clinically symptom-free animals contained no detectable ergot alkaloids in the total alkaloid EIA, and only traces (30–40 ng/g) were detected in the ergotamine EIA, close to the detection limit of the test.

No ergot alkaloids could be detected in straw and hay samples (detection limit 130 ng/g).

## Discussion

In this case series, 14 out of 103 rabbits, living under equal conditions, spontaneously showed tail lesions. Exclusively, young, growing animals were affected. As a comparable outbreak has never been reported before in scientific literature, we put some effort in identifying the likely causative factors.

We assumed that the tail lesions may be the result of peripheral ischemia due to vasoconstriction caused by mycotoxins, most likely by ergot alkaloids. In the histopathological examination, acute muscle fibre degeneration was coexisting with a chronic inflammation with granulation tissue formation. Vasculitis or thrombosis was not detectable.

The lesions observed in the deceased rabbit (hepatocellular degradation and regeneration, as well as fibrosis, bile duct hyperplasia, myocardial necrosis) could have been a possible consequence of a toxin or a toxic effect (Stalker and Hayes 2007). Such lesions are not toxin specific but have been described in association with other mycotoxins like fumonisins in swine (Colvin et al. 1993).

Considering the feed composition, the presence of *Fusarium* toxins was suspected. Indeed, *Fusarium* toxins were found in all feed samples, but at low or moderate levels in all samples. The highest levels were found for deoxynivalenol (DON) (404 ± 140 ng/g) but this can be addressed as a concentration which is commonly found in cereal-based feedstuff. The DON level was considered far too low to cause typical acute toxic effects, which are primarily characterized by feed refusal and reduced weight gain. This assumption is supported by recent data from toxicological studies (Hewitt et al. 2012) with 5-week-old New Zealand White rabbits, which suggest that rabbits are more tolerant towards DON than other species. Hewitt et al. (2012) reported that DON levels of 4.2 mg/kg in the feed, about ten times higher than those found in our study, had little effect on young fryer rabbits, only some blood parameters were slightly modified.

Although no toxicological data are available for rabbits, it can be assumed that the levels of zearalenone found in feed were also below levels which are normally involved in typical zearalenone-associated symptoms of hyperestrogenism. Furthermore, no such symptoms, which would indicate estrogenic effects, were observed. Finally, only trace amounts of fumonisins and T-2 toxin/HT-2 toxin were present in feed samples, well below levels which are regarded as toxic and also well below guidance value for fumonisins (European Commission 2006) and “indicative levels” for T-2 toxin/HT-2 toxin (European Commission 2013). Therefore, it seems unlikely that *Fusarium* toxins were associated with the observed effects.

In contrast, the results obtained for ergot alkaloids were regarded as toxicologically relevant. While hay and straw samples were free from ergot alkaloids, analysis of pelleted commercial rabbit feed yielded surprisingly high levels of ergot alkaloids in both EIAs. In Europe, rye, triticale, wheat, maize, oats and barley are the most susceptible cereals for contamination with *Claviceps* spp. Based on product specifications, the pellets fed to the rabbits contained 15 % wheat bran, 10 % peeled oat bran, 10 % wheat gluten feed and 3.1 % barley as possible sources of ergot alkaloids (Table S1, Supplementary material).

Considering factors such as analytical variability and sample homogeneity, the total ergot alkaloid content was relatively similar in most samples but a few had exceptionally higher toxin contents, with a maximum level of 1,700 ng/g (Table 3). Ergotamine seemed to be the predominant alkaloid in these samples but because of some cross-reactivity of ergocristine in the ergotamine EIA, this alkaloid may also have added to the ergotamine EIA results (Table 3). Because the mycotoxin analysis of rabbit feed started only after the clinical signs had become apparent, samples of the previous feed batches, used before November 2012, had not been available for analysis. Therefore, a clear association between ergot alkaloid content in feed and clinical symptoms could not be established.

However, as the relative feed intake of clinically affected, growing rabbits was distinctly higher (42 g per day and kg body weight) than that of healthy adult rabbits (35 g per day and kg body weight), toxic effects caused by ergot alkaloids are expected to be more pronounced in young rabbits. Using the average daily feed intake of 42 g per kg body weight of growing rabbits and the mean total ergot alkaloid content in feed (410 ng/g), the mean ergot alkaloid intake was calculated to be 17 μg per kg body weight. Using the same intake and the maximum ergot alkaloid levels found in one sample (1,700 ng/kg), the maximum daily alkaloid intake could be estimated to be 71 μg per kg body weight. However, it has to be considered that coprophagy of soft pellet faeces results in a re-ingestion and a second passage of ergot alkaloids. Therefore, the effective ergot alkaloid intake may have been much higher.

Interestingly, relatively high levels of ergot alkaloids were found in faeces from the three affected groups F1, F2 and F4. Groups F1 and F4 contained the most severely affected rabbits, while symptoms in group F2 were somewhat milder. Group F3 which was unaffected had slightly lower total ergot alkaloids in faeces, but was still positive. This is no surprise because all animals in groups F1–F4 received the same feed (Figs. 5, and 6). These findings may, speculatively, indicate that the total ingested amounts of ergot alkaloids were borderline in aspects of toxic effects. In contrast, five samples of faeces from three different groups obtained from two other private husbandries, with no clinical symptoms, were all negative for total ergot alkaloids, and only two yielded weakly positive results close to the detection limit in the ergotamine EIA.

Our hypothesis is that ergot alkaloids in rabbit faeces could possibly provide a marker for the oral intake level of mycotoxins and that the intake levels observed in this study circumscribe the toxicologically relevant dose.

Furthermore, as rabbits are a coprophagic species, ergot alkaloids in faeces undergo a recycling process, which could enhance toxin resorption. In fact, high ergot alkaloid levels were found in the faeces in the animal groups with clinical symptoms, while faeces from clinically symptom-free rabbits mostly contained no detectable alkaloids at all, and only trace amount levels were measured by the ergotamine EIA in two samples.

Gangrene of extremities (ear, tail, hoofs, combs) has been described for many animal species (Griffith et al. 1978; Diekman and Green 1992). Therefore, an exposure to ergot alkaloids seems to be a reasonable explanation for the observed symptoms. However, little is known about symptoms of acute or chronic exposure to ergot alkaloid in rabbits, and no data are available concerning the minimum effective oral dose. Therefore, our findings cannot provide clear evidence that ergot alkaloid intoxication was the cause of disease in the rabbits. Consequently, we carefully considered differential diagnoses.

Environmental and behavioural factors, such as technopathic lesions due to unsuitable housing equipment, automutilation or injuries caused by cage mates were considered but could be excluded on the basis of animal observation and after careful inspection of the housing facilities. Due to the restriction of lesions to the distal part of the tail in all animals, infections such as dermatophytosis seemed to be unlikely. Indeed, no dermatophytes were found in histological examination of PAS stained samples.

Although no indications of vasculopathy or vasculitis could be observed in the biopsies, these pathomechanisms could not be excluded. One possible aetiology for vasculitis could have been systemic infection, but neither clinical examination nor the laboratory data provided any evidence for that. The blood analysis revealed an elevated level of creatine kinase in the three affected rabbits. This was considered to be due to localized rhabdomyolysis in the tail (Aroch et al. 2010; Goto et al. 2009; Lefebvre et al. 1996). Septic diseases could also lead to an elevated level of creatine kinase because of a more generalized rhabdomyolysis (Mazaki-Tovi and Aroch 2000) but the affected rabbits never showed physical discomfort and aberrant physical parameters. No weight loss was evident in any animal during the period of observation. Because the affected rabbits were still adolescent, slightly elevated phosphorus and alkaline phosphatase levels were considered as within physiological range (Hein and Hartmann 2003). Slightly increased magnesium levels were considered as of no clinical relevance. No other immunologic stimulus was noted except vaccination, given 3 days to 3 months before the first clinical signs were observed. In dogs, ischemic dermatopathies after rabies vaccination are described (Gross et al. 2005; Vitale et al. 1999). Typical clinical signs of chronic ischemia include dry scaly skin, cutaneous atrophy and acral necrosis and resemble those lesions described here. Although vaccination as an immunological trigger for vasculitis could not be excluded, it was also regarded as unlikely because of the complete healing in most animals observed during the outbreak.

Hereditary diseases as a cause of the observed symptoms were also regarded as unlikely because the affected rabbits were offspring of four different breeding pairs.

After all these differential diagnoses had been excluded, a dietary poisoning effect was considered as the most likely cause of the observed symptoms. This is somehow supported by Griffith et al. (1978) who reported tail necrosis as a symptom of ergot alkaloid poisoning in rats. This assumption was further supported by the observation of individual animals. Animals of high rank in each group, having more frequent access to the pelleted food, were the first to show tail lesions and also were most severely affected. Likewise, young, growing rabbits with a high pellet intake had higher faecal deposits of total ergot alkaloids and ergotamine. Consequently, their dietary intake of ergot alkaloids was probably above average.

One contributing factor to the severity of the clinical symptoms could have been the prevailing climatic conditions during the outbreak. The observed onset of symptoms was on 12 November 2012. The last symptoms were observed on 07 January 2014. Therefore, all clinical cases occurred during winter 2012/2013, with average monthly temperatures of 3–5 °C and night time ambient temperatures mostly at or below 0 °C. The animals were housed under open, stable climate conditions, with sufficient shelters for protection against freezing, which is regarded as a species-appropriate condition for rabbits. Although the dose-dependent vasoconstrictory potential of ergot alkaloids remained the same, the severity of clinical effects may have been enhanced by a reduced physiological blood flow to the periphery, triggered by low ambient temperatures. It seems to be possible that neither factor alone would have caused adverse effects but that dietary ergot alkaloids plus low ambient temperature had worked synergistically.

In cattle, “fescue foot”, which is a gangrenous form of ergotism, primarily occurs during the cold season, while in summer, hyperthermia is more prevalent. Rabbits are very sensitive towards hyperthermia induced by ergot (Haschek and Voss 2013), but no information could be obtained with regard to low ambient temperature.

It seems however reasonable to assume that low ambient temperature, particularly on the floor area of the rabbit housings, may likewise have enhanced the vasoconstrictory effects of ergot alkaloids, especially in young rabbits with low body weight and high toxin intake. This would also give some explanation for the fact that the tail and not the ears was affected in young rabbits because the tail region was most likely more exposed to lower temperatures than the ears. Additionally, this would be consistent with the observation that the symptoms disappeared as soon as the rabbits reached adulthood, in spite of continuing exposure to ergot alkaloids; in adults, the ergot alkaloid-induced vasoconstriction would be less effective. It seems essential that such temperature-modulated effects should be considered in future studies on the adverse effects of ergot alkaloids in rabbits.

At present, it is unclear what the safe levels of ergot alkaloids in rabbits would be. The European Union regulations concerning ergot sclerotia in feed (European Union 2002) would correspond to a tolerance value for ergot alkaloids of 1,000 μg/kg, if an average alkaloid content of 0.1 % is assumed. The total ergot alkaloid content in most feed samples in our study was close to this level but exceeded it only in a few samples. If our assumption that ergot alkaloids in feed have caused or at least contributed to the clinical symptoms is correct, this would have practical consequences for manufacturers of commercial feedingstuff for rabbits.

To consistently avoid symptoms such as tail lesions and necrosis in younger rabbits, the mean ergot alkaloid content in such feed must be controlled and kept as low as possible. Furthermore, higher awareness of ergot-related toxicosis in rabbits may eventually yield a better overview about the overall situation in rabbit farming. Seasonal variations may be a critical factor, influencing severity of outbreaks under open-climate housing conditions, with gangrenous symptoms in the winter and possibly hyperthermia in summer.

However, to fully confirm that dietary ergot alkaloids at concentration levels of around 500 μg/kg may act as a causative agent of mycotoxicosis in rabbits, as well as to determine safe levels in feed, controlled feeding trials under various conditions would be necessary. We conclude that our results provide sufficient evidence to justify such studies, with the aim to prevent and correctly diagnose ergot-related health problems in rabbit farming.

## Electronic supplementary material

Below is the link to the electronic supplementary material.

ESM 1 (DOCX 16 kb)

ESM 2 (DOCX 14 kb)

ESM 3 (DOCX 13 kb)
