# Supplementary material for: Dietary ergot alkaloids as a possible cause of tail necrosis in rabbits
Source: Mycotoxin Res. 2014 Sep 19;30(4):241–50. doi: 10.1007/s12550-014-0208-0 (PMC4202174; doi:10.1007/s12550-014-0208-0)
Supplement: Supplementary file 1 — (DOCX 16 kb) [file 12550_2014_208_MOESM1_ESM.docx]

**Supplementary material**

**Table 1: Declaration „HasFit Carat“ Pellets, 25 kg bag, batch 1**

| **Composition** | **%** |
| --- | --- |
| Lucerne meal | 22 |
| Wheat bran | 15 |
| Sunflower seed, extracted | 14 |
| Wheat | 10 |
| Peeled oat bran | 10 |
| Wheat gluten feed | 10 |
| Soy extraction grist | 3.3 |
| Dried beet pulp | 3.3 |
| Barley | 3.1 |
| Rapeseed, extracted | 2.5 |
| Sugar beet pulp | 1.8 |
| Calcium carbonate | 0.70 |
| Vegetable oil (palm, rape, sunflower oil) | 0.5 |
| Sodium chloride | 0.32 |
| **Analytical constituents and levels** | **%** |
| Crude protein | 17 |
| Crude fat | 3.5 |
| Crude ash | 7.5 |
| Crude fiber | 15 |
| Calcium | 1 |
| Phosphorus | 0.55 |
| **Nutritional additives per kg** | **I.U.** |
| Vitamin A | 12000 |
| Vitamin D3 | 1200 |
|  | **mg** |
| Vitamin E | 55 |
| Copper (Copper-II-sulphate pentahydrate) | 12 |
| Selenium (Sodium selenite) | 0.3 |
| Zinc (Zinc-sulphate monohydrate) | 80 |
| Iron (Iron-sulpahte monohydrate) | 115 |
| Manganese (Manganese-II-sulphate monohydrate) | 80 |
| Iodine (Calcium iodate, anhydrous) | 1 |
| **Technological additives** |  |
| Formic acid |  |
| Ammonium formiate |  |
| Propionic acid |  |
