# Supplementary material for: Dietary ergot alkaloids as a possible cause of tail necrosis in rabbits
Source: Mycotoxin Res. 2014 Sep 19;30(4):241–50. doi: 10.1007/s12550-014-0208-0 (PMC4202174; doi:10.1007/s12550-014-0208-0)
Supplement: Supplementary file 2 — (DOCX 14 kb) [file 12550_2014_208_MOESM2_ESM.docx]

**Table 2: Biochemistry profile of affected (82050, 82061, 82063) and non affected rabbits (82053, 82076)**

|  |  | **affected rabbits** |  |  | **non affected rabbits** |  |
| --- | --- | --- | --- | --- | --- | --- |
|  |  | **82050** | **82061** | **82063** | **82053** | **82076** |
|  |  | **m** | **f** | **f** | **f** | **f** |
| **Parameter (in house reference)** |  |  |  |  |  |  |
| **UREA (4.83-8.96 mmol/l)** |  | 5.5 | 6.62 | 6.86 | 6.08 | 7.28 |
| **CREA (34-166μmol/l)** |  | 79 | 63 | 115 | 109 | 61 |
| **Na ion. (133-143 mmol/l)** |  | 138 | 136 | 139 | 135 | 137 |
| **Cl ion. (93-109 mmol/l)** |  | 93 | 97 | 94 | 95 | 93 |
| **K ion. (3.7-6.3 mmol/l)** |  | 4.81 | 4.6 | 4.14 | 3.89 | 4.06 |
| **Ca ion. (1-1.79 mmol/l)**  **Phos (1.02-1.97 mmol/l)**  **Mg ion. (0.53-0.65 mmol/l)**  **TP ( 51.94-72.02 g/l)** |  | 1.66  2.34  0.74  63.5 | 1.7  2.31  0.74  56.2 | 1.56  2.23  0.72  62.4 | 1.76  2.11  0.74  63.5 | 1.72  2.41  0.74  60.3 |
| **ALB (34-42.7 g/l)**  **GLO (15-32 g/l)**  **GLUC (5.8-14.8 mmol/l)**  **BILIT (0.29-2.53 µmol/l)**  **CHOL (0.5-4.8 mmol/l)** |  | 41.9  21.6  8.71  1.61  1.09 | 37.8  18.4  9.33  2.13  1.4 | 44.1  18.3  9.97  1.62  1.31 | 39.7  23.8  9.09  1.89  1.58 | 43.8  16.5  9.39  1.22  2.03 |
| **TRI (0.5-3.4 mmol/l)** |  | 1.63 | 0.59 | 0.94 | 1.02 | 1.3 |
| **ALP (< 132 U/l)** |  | 242 | 148 | 165 | 210 | 244 |
| **ALT (0-87 U/l)** |  | 77 | 65 | 64 | 77 | 90 |
| **GLDH ( < 27,3U/l)** |  | 5 | 5 | 6 | 8 | 6 |
| **CK (< 1331 U/l)** |  | 1465^*^ | 2131^*^ | 1944^*^ | 617 | 744 |

m=male, f=female, **^*^**p ≤ 0.05

UREA= urea, CREA=creatine, Na ion.=ionised sodium, Cl ion.=ionised chloride, K ion.= ionised potassium, Ca ion.= ionised calcium, Phos= phosphorus, Mg ion.= ionised magnesium, TP= total protein, ALB= albumin GLO= globulin, GLUC= glucose, BILIT= total bilirubin, CHOL= cholesterol, TRI= triglyceride, ALP= alkaline phosphatase, ALT= alanine aminotransferase, GLDH= glutamate dehydrogenase, CK= creatine kinase
