# Supplementary material for: Dietary ergot alkaloids as a possible cause of tail necrosis in rabbits
Source: Mycotoxin Res. 2014 Sep 19;30(4):241–50. doi: 10.1007/s12550-014-0208-0 (PMC4202174; doi:10.1007/s12550-014-0208-0)
Supplement: Supplementary file 3 — (DOCX 13 kb) [file 12550_2014_208_MOESM3_ESM.docx]

**Table 3: Haematology of affected (82050, 82061, 82063) and non affected rabbits (82053, 82076)**

|  | **affected rabbits** |  |  | **non affected rabbits** |  |
| --- | --- | --- | --- | --- | --- |
|  | **82050** | **82061** | **82063** | **82053** | **82076** |
|  | **m** | **f** | **f** | **f** | **f** |
| **Parameter (in house reference)** |  |  |  |  |  |
| **WBC ( 5.65-16.67 10^9^/l)** | 5.41 | 9.86 | 6.84 | 4.87 | 6.71 |
| **NEU (1.36-9.31 10^9^/l)** | 1.27 | 1.88 | 1.66 | 1.00 | 1.31 |
| **LYM ( 2.8-7.2 10^9^/l)** | 3.48 | 7.24 | 4.59 | 3.26 | 5.07 |
| **MON (0.16-0.79 10^9^/l)** | 0.07 | 0.11 | 0.18 | 0.1 | 0.05 |
| **EOS (0.13-0.61 10^9^/l)** | 0.13 | 0.31 | 0.23 | 0.05 | 0.14 |
| **BASO (0-0.55 10^9^/l)** | 0.08 | 0.3 | 0.14 | 0.43 | 0.11 |
| **RBC (5.77-7.34 10^12^/l)**  **HTC (0.36-0.48 l/l)** | 5.31  0.33 | 6.68  0.4 | 6.42  0.42 | 5.75  0.36 | 6.06  0.4 |
| **HB (7-10.6 mmol/l)** | 7.7 | 9.1 | 9.3 | 8.5 | 9.1 |
| **MCV ( 59.3-69.6 fl)** | 62.6 | 59.4 | 65.2 | 62.8 | 65.3 |
| **MCH (1.35-1.62 fmol/l)** | 1.46 | 1.36 | 1.45 | 1.48 | 1.5 |
| **MCHC (18.4-20.1 mmol/l)** | 23.29 | 22.87 | 22.21 | 23.51 | 22.95 |
| **PLT (276-769 10^9^/l)** | 274 | 500 | 495 | 559 | 404 |

m=male, f=female

WBC= white blood cells, NEU= neutrophile granulocytes, LYM= lymphocytes, MON= monocytes, EOS= eosinophile granylocytes, RBC= red blood cells, HTC= hematocrit, HB= hemoglobin, MCV= mean corpuscular volume, MCH= mean corpuscular hemoglobin, MCHC= mean corpuscular hemoglobin concentration, PLT= platelets
